# Wnt/β-Catenin Protects Lymphocytes from HIV-Mediated Apoptosis via Induction of Bcl-xL

**DOI:** 10.3390/v14071469

**Published:** 2022-07-02

**Authors:** Yasmeen A. Albalawi, Srinivas D. Narasipura, Lena Al-Harthi

**Affiliations:** 1 Department of Microbial Pathogens and Immunity, Rush University Medical Center, Chicago, IL 60612, USA; yasmeen_a_albalawi@rush.edu (Y.A.A.); srinivasa_narasipura@rush.edu (S.D.N.); 2 Department of Biology, College of Science, Jouf University, Sakaka 72341, Aljouf, Saudi Arabia

**Keywords:** HIV, apoptosis, Wnt/β-catenin pathway, CD4 T cells, CD4^dim^CD8^bright^ T cells, Bcl-xL

## Abstract

HIV infection mediates the apoptosis of lymphocytes, the mechanism of which is multifaceted. Here, we evaluated the role of Wnt/β-catenin signaling in HIV-associated T cell apoptosis, as Wnt/β-catenin regulates the transcriptional activity of genes impacting apoptosis. We specifically investigated the role of the Wnt/β-catenin pathway in the HIV-associated apoptosis of CD4+ T cells and CD4^dim^CD8^bright^ T cells, a population that is infected by HIV. We found that the induction of β-catenin, via a 6-bromoindirubin-3-oxime (BIO), significantly rescued HIV-infected CD4+ and CD4^dim^CD8^bright^ T cells from apoptosis by >40–50%. Further, a small-molecule inhibitor of the Wnt/β-catenin pathway (PNU-74654) reversed BIO-mediated protection from HIV-associated apoptosis. BIO also induced Bcl-xL, an anti-apoptotic protein, and a target gene of Wnt/β-catenin, in CD4+ and CD4^dim^CD8^bright^ T cells by approximately 3-fold. Inhibiting Bcl-xL by WEHI-539 abrogated β-catenin-mediated apoptotic protection in infected CD4+ and CD4^dim^CD8^bright^ T cells. Collectively, these findings demonstrate that engaging Wnt/β-catenin signaling in HIV-infected T cells protects them from HIV-associated apoptosis by inducing Bcl-xL.

## 1. Introduction

The canonical Wnt/β-catenin pathway is a pro-survival pathway that is robustly expressed in many tissues and organ systems. β-catenin is the central mediator and transcriptional coactivator of this pathway. The pathway is induced by evolutionarily conserved Wnt ligands (19 small secreted glycoproteins) that bind to a seven-transmembrane frizzled receptor and co-receptor (low-density lipoprotein-receptor-related protein (LRP 5/6)), leading to the destabilization of the proteasomal destruction complex, the hypo-phosphorylation of β-catenin, and its translocation to the nucleus, where it binds to members of the T cell factor (TCF)/lymphoid enhancer (LEF) family of transcriptional factors to regulate target gene expression [1,2]. These genes are involved in proliferation, wound healing, survival, development, stress, regeneration and stemness. β-catenin, additionally, is an essential component of the adheren junction protein complex, which connects N-cadherin, α-catenin, and actin and regulates cell-to-cell communication [2,3,4,5].

Emerging data point to the impact of Wnt/β-catenin on several neurotropic viruses. Specifically, in response to West Nile virus and influenza virus infection, adherens, tight-junction proteins (ZO-1, claudin-1, occludin), and β-catenin are disrupted in brain endothelial cells, leading to increased viral infiltration into the CNS [6,7,8]. In the context of HIV, β-catenin is a repressor of HIV transcription [9,10]. Particularly, β-catenin binds to TCF-4 on the HIV promoter at −336, −143, +66, and +186, with the strongest association occurring at −143, whereby a multiprotein complex is formed between β-catenin; TCF-4; and the nuclear matrix protein, SMAR, to pull the HIV LTR away from the transcriptional machinery disrupting HIV transcription [10]. Further, β-catenin regulates the expression of CD4 on CD8+ T cells to generate a unique T cell phenotype known as CD4^dim^CD8^bright^ T cells [11]. These cells are elevated among HIV long-term non-progressors and are enriched in anti-HIV cytolytic activity [12]. BcL-xL, a member of the BCL-2 family of proteins, is enriched in CD4^dim^CD8^bright^ T cells. Similarly, Bcl-xL is upregulated in CD4+CD8+ (double positive) thymocytes to promote their survival, while thymocytes lacking Bcl-xL die prematurely [13,14]. The stabilization of β-catenin signaling in double-positive thymocytes leads to the activation of the downstream target gene Bcl-xL and protection from apoptosis. β-catenin directly activates Bcl-xL promoter activity and modulates it at the transcriptional level [15]. Furthermore, the BCL-2 protein family plays a significant role in determining cell fate by either promoting or inhibiting apoptosis [16]. BCL2, Bcl-xL (gene/transcript name BCL2L1), MCL1, BCL2A1, and BCL-W are anti-apoptotic proteins which promote cell survival [17]. BAX and BAK, on the other hand, are pro-apoptotic and are essential for the execution phase of the mitochondrial apoptosis pathway [18]. Cell fate is determined by the balance between these anti-apoptotic and pro-apoptotic members.

Understanding the pathways by which HIV causes infected and non-infected cells to die can inform targeted therapeutics to engage these pathways to protect the cells from HIV direct and indirect killing or, conversely, to mediate the killing of latently infected cells. Approximately 5% of dying cells are productively infected with HIV, while the majority of cell death occurs in “bystander” cells—those which have undergone abortive infection and aberrant apoptosis [19,20,21]. The direct or bystander HIV-mediated cell death of CD4+ T cells is attributed to apoptosis, pyroptosis, and autophagy (autolysis) [19,20,21]. These mechanisms are triggered by a variety of stimuli, including immune cell overexpression of death ligands (TNF, Fas Ligand and TRAIL) [22,23]; direct cytotoxicity of a number of proteins such as gp120, Nef, Tat, Vpu, and Vpr [24,25]; and/or activation-induced cell death (AICD) [26]. HIV-infected cells largely undergo caspase-3-mediated apoptosis. In contrast, bystander killing due to abortive HIV infection leads to the accumulation of incomplete cytosolic viral DNA transcripts identified by IFI16 [19,27]. This triggers an inflammasome, which activates caspase-1 and leads to pyroptosis [28]. Pyroptosis occurs only during cell-to-cell contact, so the virus will spread, while cell-free virions will fail to trigger the same response [28].

Given that β-catenin is a pro-survival factor, on the one hand, and an inhibitor of HIV transcriptional activity on the other, we assessed here whether β-catenin can protect HIV-infected cells from apoptosis in both CD4+ T cells and CD4^dim^CD8^bright^ T cells. The long-term survival of these infected cells can contribute to HIV latency in these populations. Using specific inhibitors, we show that β-catenin protects HIV-infected lymphocytes from apoptosis through the induction of its downstream target, Bcl-xL.

## 2. Material and Methods

**Ethics statements:** Research involving human subjects was conducted in accordance with institutional (IRB-L06080703) and U.S. government guidelines on human research. Whole blood was collected from healthy HIV-seronegative donors at Rush University Medical Center. Written informed consent was obtained from all participants prior to blood donation, and this study was approved by the Institutional Review Board of Rush University Medical Center.

**Cell culture:** Peripheral blood mononuclear cells (PBMCs) were isolated by the Ficoll–Hypaque density gradient centrifugation of venous blood collected from healthy individuals. PBMCs were suspended in complete RPMI 1640 medium (Thermo Fisher Scientific, Waltham, MA, USA), which includes 10% heat-inactivated FBS (Sigma, St. Louis, MO, USA), 1% penicillin/streptomycin (ThermoFisher), and 2 mM L-glutamine (GlutaMAX Supplement, ThermoFisher). Cultures were stimulated with 30 U/mL IL-2 (AIDS Reagent Program, Germantown, MD, USA) and 1 µg/mL soluble anti-CD3/anti-CD28 Abs (BD Biosciences, San Jose, CA, USA) and maintained for 3 days.

**HIV infection and moiety of infection (MOI):** PBMCs were infected with HIV_BaL_ at 2 ng (MOI of 0.2) p24/1 × 10^6^ cells for 6 h at 37 °C, 5% CO_2_. Subsequently, the unbound virus was removed by thoroughly washing the cells. The cells were then cultured in complete RPMI supplemented with 30U/mL of IL-2. HIV replication was monitored for 6 days post-infection by harvesting some cells and measuring the intracellular HIV-1 core p24 Ag by flow cytometry. MOI of HIV_Bal_ was determined using the TZM-bl reporter cell line (NIH AIDS Reagent program #8129) seeded in 24-well flat bottom plates. Cells were infected with 2ng of HIV_Bal_ and the activity of β-galactosidase was measured 48 h post infection using the β-Gal Staining Kit according to the manufacturer’s instructions (Thermo Fisher Scientific, Waltham, MA, USA).

**Induction and inhibition of β-catenin in PBMCs**: In some experiments, 6-bromoindirubin-3′-oxime (6-BIO) (Enzo life sciences, Farmingdale, NY, USA) was added at a 5 µM concentration post infection, and cells were cultured at 37 °C in 5% CO_2_. PNU-74654 (Enzo life sciences), a β-catenin inhibitor, was added to cells at a 50 µM concentration post-infection. Cells were treated with WEHI-539 (ApexBio, Houston, TX, USA), a Bcl-xL inhibitor, at 5 µM concentration post infection, and cultured to day 6, then washed and stained for flow cytometry and apoptosis analysis. All the drugs were re-suspended in an appropriate vehicle and stored per the manufacturer’s instructions.

**Cell viability and apoptosis:** Cell viability was monitored using the trypan blue exclusion assay. The level of apoptosis was evaluated on day 6 post infection by an annexin V/propidium iodide (PI) flow-based assay using annexin V apoptosis detection kit I, as per the manufacturer’s instructions (BD Biosciences). Cells were first extracellularly stained with CD3- Pacific Blue (clone SP34-2), CD4-APC (clone RPA-T4), and CD8-APC-H7 (clone SK1) (BD Biosciences), followed by staining for annexin-V-FITC and PI according to the manufacturer’s instructions. Flow data were collected on the FACS Canto II flow cytometer with the BD FACSDiva software (BD Biosciences) and analyzed using the FlowJo software (BD Biosciences).

**Flow cytometric analysis:** On day 6 post infection, PBMCs were surface-stained with CD3- Pacific Blue (clone SP34-2), CD4-APC (clone RPA-T4), and CD8-APC-H7 (clone SK1). For the intracellular detection of HIV-1 core Ag p24 or Bcl-xL, the cells were fixed and permeabilized using Cytofix/Cytoperm (BD Biosciences), according to the manufacturer’s instructions, before staining with 5 µL KC57-RD1 (PE) (clone KC57) or Bcl-xL monoclonal antibody (PE) (clone 7B2.5) (ThermoFisher). Lymphocytes singlet events were collected in each sample and analyzed to maintain cell number between samples for the comparison of different T cell populations. Data were collected on an LSRFortessa flow cytometer with the BD FACSDiva software and analyzed using the FlowJo software.

**Transfection with TOPflash and dual luciferase assay**: To measure β-catenin–dependent signaling activity, 5 × 10^6^ PBMCs cultured in complete RPMI were transfected with 10 μg TOPflash reporter construct (Millipore, Billerica, MA, USA), using the P3 Primary Cell 4D-NucleofectorTM X Kit L (Lonza, BioWhittaker, Walkersville, MD, USA) and 4D-Nucleofector Core Unit Catalog #: AAF-1002B (Lonza, BioWhittaker, Walkersville, MD, USA), as recommended. Cells were recovered in complete RPMI with 30 U/mL IL-2 for 2 h. Subsequently, cells were treated with BIO (5 µM) or vehicle (DMSO). Forty-eight hours post transfection, plasmid reporter activity was performed with a dual-luciferase reporter assay using 10–20 μL lysate (Promega, Madison, WI, USA), using a Sirius Single tube luminometer (Berthold Detection Systems, Pforzheim, Germany). The total protein concentration was measured by a Pierce bicinchoninic acid (BCA) protein assay kit (Thermo Fisher Scientific) and data were represented as relative luciferase units (RLU)/µg of protein.

**Statistical analysis:** Statistical analyses were performed using the Prism software (GraphPad Prism, San Diego, CA, USA). The variables were compared using either a one-way analysis of variance (ANOVA) and Newman–Keuls multiple comparisons test or a two-tailed Student’s *t* test, as indicated. All experiments were performed independently at least three times and data were represented as means, medians, or fold changes with the standard error of mean (SEM), with *p* ≤ 0.05 considered statistically significant.

## 3. Results

**Figure 1:**
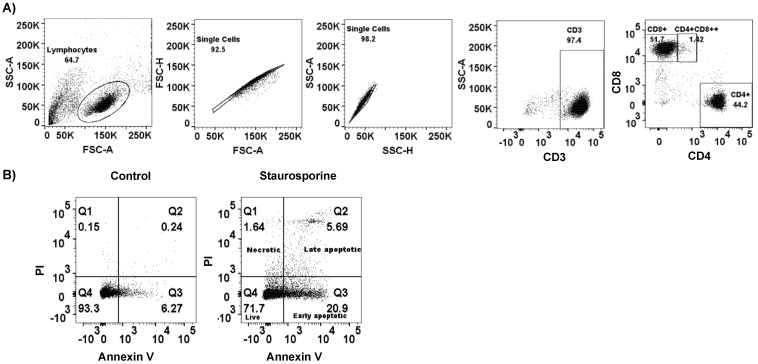

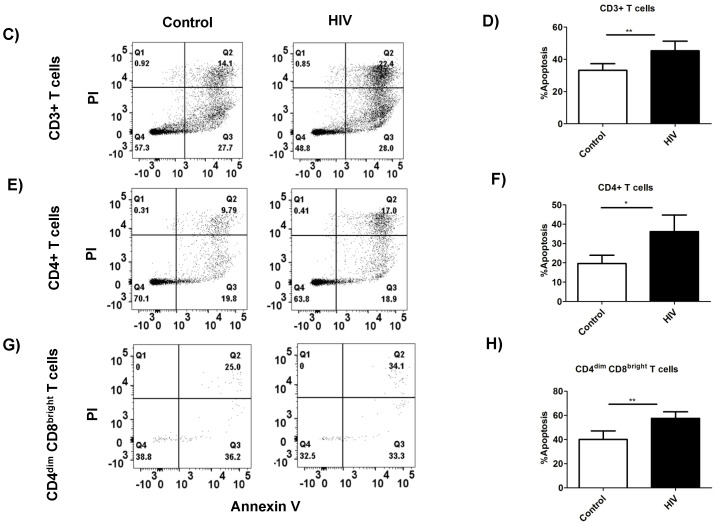
HIV infection induces apoptosis-mediated cell death in CD3+, CD4+, and CD4^dim^CD8^bright^ T cells. (**A**,**B**) PBMCs were cultured in complete RPMI and activated for 3 days in the presence of 30 U/mL IL-2 and then treated with staurosporine (1 µM) for 24 h. (**A**) Gating strategy for lymphocytes was based on side scatter (SSC-A) versus forward scatter (FSC-A). Doublets were excluded by gating for FSC-A vs. FSC-H then SSC-A vs. SSC-H. T cells were gated on the basis of the surface expression of CD3 and further broken down into CD8 single positive (CD8^bright^ CD4^−^), CD8 double positive (CD4^dim^CD8^bright^), or CD4 single positive (CD4^bright^ CD8^−^), as shown. (**B**) General gating strategy to measure apoptosis and/or necrosis by an annexin/PI flow-based assay in each cell population, as shown in representative dot plots. Left to right, control, staurosporine (1 µM). (**C**–**H**) Three-day-activated PBMCs were infected with HIV_Bal_ for 6 h or left uninfected, washed, and cultured in complete RPMI for 6 days. (**C**,**E**,**G**) Representative dot blots of day six post infection or control (non-infected) cells showing annexin V positive populations in CD3+, CD4+ and CD4^dim^CD8^bright^ T cells, respectively. (**D**,**F**,**H**) Cumulative data of percent apoptosis (early and late apoptosis) on day six post infection and control in CD3, CD4+ and CD4^dim^CD8^bright^ T cells populations. *n* = 4 per treatment. Data are represented as mean ± SEM and analyzed using one-way ANOVA and Newman–Keuls multiple comparisons test. * means *p* ≤ 0.05, ** *p* ≤ 0.01.

**HIV infection induces apoptosis in CD4+ andCD4^DIM^CD8^BRIGHT^ T cells:** To determine the extent of HIV-induced cell death, we infected activated PBMCs with HIV and measured apoptosis 6 days post infection using an annexin/PI flow-based assay. The gating strategies used on lymphocyte, singlet, CD3+, CD4+, and CD4^dim^CD8^bright^ T cells were as shown in Figure 1A. Staurosporine (1 µM), an apoptosis-inducing agent through caspase-dependent and -independent pathways [29], was used as a positive control to set gates on live, early, late apoptotic, and necrotic populations (Figure 1B and Figure S1A). Staurosporine at 1 µM exhibited a wide range of killing (25–75%) in activated PBMCs, which could be attributed to donor variability (Figure S1A). By measuring the percentages of total apoptosis (early and late apoptosis), we found that approximately 30–50% of total CD3+ T cells, 30–45% of CD4+ T cells, and 30–70% of CD4^dim^CD8^bright^ T cells were positive for apoptosis in the presence of HIV, which was significantly higher than the basal level in uninfected control populations (Figure 1C–H). To further confirm the effect of HIV infection on PBMCs, we evaluated the ratios of CD4–CD8 T cells in PBMCs before and after infection. The CD4 percentage was downregulated post HIV infection, although it did not reach statistical significance, while CD8+ and CD4^dim^CD8^bright^ T cells percentages increased post infection (Figure S1B–E). Interestingly, by dividing the apoptosis process into early and late stages, we found no significant difference in the percentages of early apoptosis before and after HIV infection in all T cell populations (Figure S2A–C). However, the percentages of late apoptosis were significantly higher as a result of HIV infection in both CD3+ and CD4+ T cells and, although not significant, a trend in induction was observed in in CD4^dim^CD8^bright^ T cells (Figure S2D–F).

**Figure 2:**
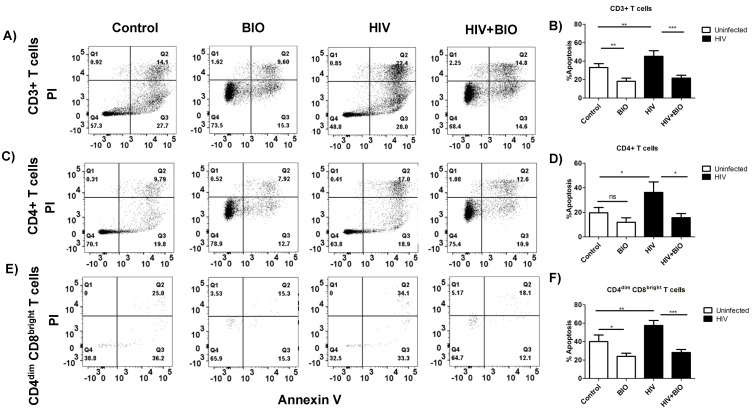
Induction of Wnt/β-catenin signaling protects T cell subsets from HIV-mediated apoptosis. PBMCs were cultured in complete RPMI and activated for 3 days with anti-CD3/CD28 antibodies in the presence of 30 U/mL IL-2, then infected with HIV_Bal_, or left uninfected, for 6 h, washed, and 24 h. post infection cells were treated with BIO or DMSO. (**A**,**C**,**E**) Representative dot blots of day six post infection and treatment with BIO in CD3, CD4+, and CD4^dim^CD8^bright^ T cell populations. Flow data were collected and analyzed. (**B**,**D**,**F**) Cumulative data of apoptosis percentage (early and late apoptosis) on day six post infection and treatment with BIO in CD3+, CD4+, and CD4^dim^CD8^bright^ T cell populations. *n* = 4 per treatment. Data are represented as mean ± SEM and analyzed using one-way ANOVA and Newman–Keuls multiple comparisons test. * means *p* ≤ 0.05, ** *p* ≤ 0.01, *** *p* ≤ 0.001. ns denotes no significance.

**Induction of Wnt/β-catenin signaling protects T cell subsets from HIV-mediated apoptosis:** To determine the impact of β-catenin on protecting T cell subsets from HIV-mediated apoptosis, we treated PBMCs with a 6-bromoindirubin-3-oxime (BIO), a small-molecule inducer of β-catenin which inhibits GSK-3β, a component of the β-catenin destruction complex. By inhibiting GSK3β, β-catenin is hypophosphorylated and not tagged for proteasomal degradation. As expected, transfecting PBMCs with Topflash and treating with BIO for 48 h significantly induced the luciferase reporter activity by 3–5-fold when compared with vehicle (DMSO) treatment (Figure S3A). PBMCs infected with HIV and treated with BIO demonstrated a significant reduction in total apoptosis by >40–50% in CD3+, CD4+, and CD4^dim^CD8^bright^ T cells (Figure 2A–F). Interestingly, when divided into early and late apoptotic stages, both stages were found to be significantly rescued as a result of β-catenin induction in the absence or presence of HIV infection in CD3+ and CD4+ T cell populations (Figure S3B,C,E,F). However, in the CD4^dim^CD8^bright^ T cell population, although not significant, there was a trend of rescue in the early stage (Figure S3D) which was not evident in the late stage (Figure S3G). Cumulatively, these data demonstrate that the induction of β-catenin signaling significantly protects CD4+ T lymphocytes from HIV-mediated apoptosis.

**Figure 3:**
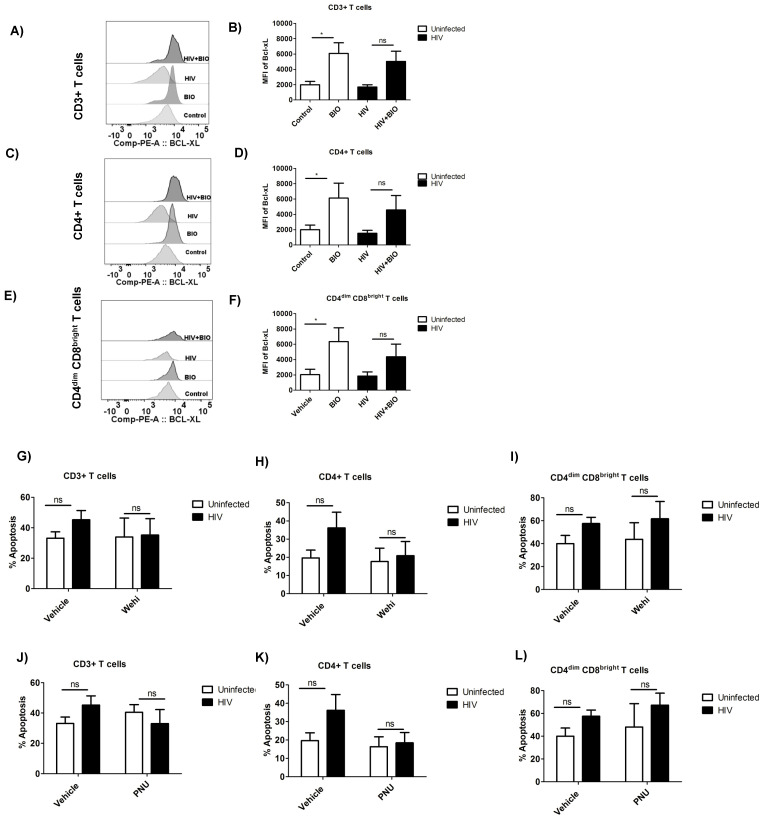
β-catenin protects CD4+ and CD4^dim^CD8^bright^ T cells from HIV-induced apoptosis through the induction of Bcl-xL. PBMCs were cultured in complete RPMI and activated for 3 days with anti CD3/CD28 antibodies in the presence of 30 U/mL IL-2, then infected with HIV_Bal_, or left uninfected, for 6 h and washed. Cells 24 h post infection were treated with BIO or DMSO. Mean fluorescence intensity (MFI) of Bcl-xL was measured on day 6 post infection in CD3, CD4+, and CD4^dim^CD8^bright^ T cells. (**A**,**C**,**E**) Representative flow histograms of MFI of Bcl-xL in CD3+, CD4+, and CD4^dim^CD8^bright^ T cells, respectively. (**B**,**D**,**F**) Cumulative data of Bcl-xL MFI, as measured on day 6 post infection in CD3, CD4+, and CD4^dim^CD8^bright^ T cells. *n* = 5 per group. Data are represented as mean ± SEM and analyzed using one-way ANOVA and Newman–Keuls multiple comparisons test. * means *p* ≤ 0.05. (**G**–**L**) PNU-7465 and WEHI-539 do not contribute to apoptosis**.** Three-day-activated PBMCs were infected with HIV_Bal_ for 6 h, or left uninfected, and washed. Twenty-four hours post infection, cells were treated with PNU-74654 (50 µM) or WEHI-539 (5 µM) or proper vehicle control; 6 days post infection, apoptosis was measured using flow cytometry. Cumulative data of mean percentage of total apoptosis in CD3+, CD4+, and CD4^dim^CD8^bright^ T cell populations were represented. *n* = 4 per treatment. Data are represented as mean ± SEM and analyzed using one-way ANOVA and Newman–Keuls multiple comparisons test. * means *p* ≤ 0.05. ns denotes no significance.

β-catenin protects CD4+ and CD4^dim^CD8^bright^ T cells from HIV-induced apoptosis through the induction of the anti-apoptotic protein Bcl-xL. We next assessed whether Wnt/β-catenin protection from HIV-mediated apoptosis is, through its effect on induction of Bcl-xL, an anti-apoptotic downstream target gene of Wnt/β-catenin signaling. We show that exposure to BIO-induced Bcl-xL protein expression, as measured by intracellular flow cytometry in CD3+ T cells by ~3-fold as well as on CD4+ T cells and CD4^dim^CD8^bright^ T cells; this induction was also evident post-HIV infection (Figure 3A–F). Interestingly, in the HIV-infected population, the MFI of Bcl-xL was slightly reduced only in CD4+ T cells, while it maintained its levels in the CD3+ population and CD4^dim^CD8^bright^ T cells when compared with their respective uninfected populations (Figure 3B,D,F).

**Figure 4:**
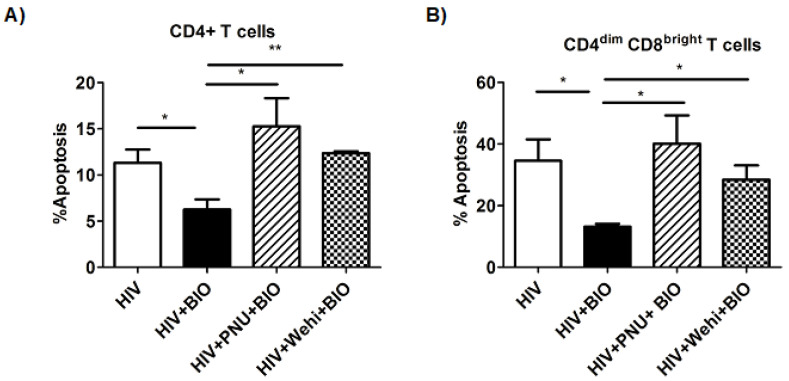
PNU-7465 (specific Wnt/β-catenin pathway inhibitor) and WEHI-539 (specific Bcl-xL inhibitor) reverses mediated protection by Wnt/β-catenin against HIV-induced cell death in T lymphocytes. Three-day-activated PBMCs were infected with HIV_Bal_ for 6 h, washed and treated with 5 µM BIO, PNU-74654 at 50 µM, or WEHI-539 at 5 µM or vehicle after 24 h of infection. (**A**,**B**) Cumulative data of apoptosis mean percent on day 6 post infection and treatment with PNU-7465 and WEHI-539 in CD4+ and CD4^dim^CD8^bright^ T cell populations. Flow data were collected on a FACS Canto II flow cytometer with the BD FACSDiva software (BD Biosciences, Franklin Lakes, NJ, USA) and analyzed using the FlowJo software. Data are represented as mean ± SEM and analyzed using a two-tailed one-sample *t* test. * means *p* ≤ 0.05, ** *p* ≤ 0.01.

We next evaluated whether the inhibition of Bcl-xL would impact HIV-mediated apoptosis. We used WEHI-539, a specific small-molecule inhibitor of Bcl-xL, which interacts with Bcl-xL binding groove to inhibit its anti-apoptotic action [30]. Treatment with WEHI-539 alone had no effect on the apoptosis rate of infected or uninfected cells (Figure 3G–I). HIV-infected cells treated with BIO and WEHI-539 abrogated the protective effect of BIO restoring the rate of apoptosis to the basal uninfected level in both CD4+ and CD4^dim^CD8^bright^ T cells (Figure 4A,B).

To rule out the possibility that apoptosis reversal can occur due to the inhibition of GSK3β via BIO, we used an inhibitor of Wnt/β-catenin pathway activity, PNU-74654, which is known to inhibit the interaction between β-catenin and TCF [31]. The inhibitor by itself had no effect on cell death or apoptosis when compared to controls (DMSO) in the presence or absence of HIV (Figure 3J–L). However, in BIO-treated cells, PNU-74654 reversed the protective effect of BIO by increasing the apoptosis close to the levels of HIV-infected control cells in CD4+ T cells (Figure 4A), as well as in CD4^dim^CD8^bright^ T cells (Figure 4B).

## 4. Discussion

Significant progress has been made in understanding the mechanisms by which HIV-1 triggers CD4+ T cell death, which is progressively depleted in the course of the natural history of HIV infection, prior to advances in Combined Antiretroviral Therapy (cART) [32]. In this study, we assessed whether the induction of β-catenin signaling can protect cells from HIV-associated T-cell death. We focused on two populations, CD4+ T cells, which are the predominant cell type infected by HIV, and CD4^dim^CD8^bright^ T cells, a unique population that is also infected with HIV and mediates robust anti-HIV cytotoxic T cell responses [12]. We found that the induction of β-catenin in both of these populations rescued them from HIV-mediated cell death. By utilizing specific small-molecule inhibitors (PNU-74654 and WEHI-539), we show that this protection is mediated through the β-catenin induction of Bcl-xL, a Wnt/β-catenin downstream anti-apoptotic gene target. Our findings highlight the significant role of canonical Wnt signaling in protecting against HIV-associated T cell apoptosis. It is worth noting that the induction of β-catenin also protected the non-HIV infected cells from apoptosis under normal conditions. Further, we recently showed that β-catenin signaling drives CD4 expression on CD8 T cells and that they are enriched in its target anti-apoptotic gene Bcl-xL [11]. The induction of β-catenin through Bcl-XL protects CD4^dim^CD8^bright^ T cells from HIV-associated apoptosis. Furthermore, although we showed that antagonizing Bcl-xL can mediate the apoptosis of HIV-infected cells, our study was not designed to assess the difference between those that are productively infected, latently infected, or those that die through bystander killings associated with HIV infection.

During acute and chronic HIV infection, CD4 T cells are lost primarily by activation-induced cell death, culminating in apoptosis. We used a flow cytometry-based assay to measure apoptosis via Annexin V Staining; fluorescent annexin V conjugates are used to identify apoptotic cells. Annexin V is a recombinant Ca^2+^ dependent phospholipid-binding protein with a high affinity for the anionic phospholipid phosphatidylserine (PS). PS is found on the cytoplasmic surface of the plasma membrane in healthy cells. The plasma membrane, on the other hand, undergoes structural changes during apoptosis, including the translocation of PS from the inner to extracellular side of the plasma membrane. The translocated PS on the cell’s outer membrane tags the cell for identification and phagocytosis by macrophages [33]. This assay is considered highly sensitive, since it can detect apoptosis on a single cell level; however, necrotic cells can be labeled as well. Adding membrane-impermeant nucleic acid dyes such as propidium iodide stains necrotic cells, since it has the ability to detect loss of membrane integrity as a pathognomonic feature. Using this flow-based assay, we were unable to discriminate between the apoptosis of productively infected cells and bystander cells for technical reasons. Propidium Iodide (PI) and p24 gag-PE fall in the same laser emission spectrum. Although p24-gag conjugated to FITC is commercially available, Annexin V is also conjugated to FITC, again including both antibodies within the same flow assay. Further, intracellular staining for p24-gag requires the intracellular fixation and permeabilization of the cells which will compromise the plasma membrane and Annexin V apoptosis assay. It is worth mentioning that HIV is not only involved in pro-apoptotic pathways. On the contrary, there is growing evidence to support the anti-apoptotic effects of HIV on infected cells. This will allow the infected host cells to avoid the immune response, either by inducing protection against various death ligands expressed by effector cells or by avoiding the detection of infected cells by pattern recognition receptors (PRRs). Such cellular effects can benefit the virus to establish latency, reactivation, and viral replication [34].

β-catenin is a repressor of HIV transcription, and we show here that it protects cells from HIV-associated apoptosis. These two effects are likely to promote HIV latency in infected cells. Previous data from our lab have identified Wnt/β-catenin signaling as a restriction factor for HIV in a number of cell types, including PBMCs, astrocytes, and monocyte/macrophages [9,10,35,36,37]. β-catenin can inhibit HIV replication through binding to TCF-4 on the HIV LTR and interfering with HIV transcription [10,37,38]. Being at this interface of repressing HIV replication and also a pro-survival molecule, the induction of β-catenin can potentially lead to a population of T cells that are HIV-infected yet are not dying, facilitating a reservoir of HIV that can be latent. Therapies that activate β-catenin, on the one hand, can suppress HIV transcription and also promote the survivability of both infected and non-infected cells, while those that suppress β-catenin can lead to the activation of HIV and the enhanced apoptosis of HIV-infected cells. This can be an advantage to shock and kill or block and lock in HIV cure strategies. In shock and kill, the goal is to activate HIV from latency by targeting the cellular pathways responsible for HIV latency [38], whereas in block and lock, the goal is to permanently maintain HIV dormancy in infected cells without antiretroviral therapy [39].

Recently, antagonizing Bcl-xL in a cell model of HIV latency demonstrated the elimination of infected cells, yet this was not translated to CD4+ T cells from cART-suppressed donors [40]. This shortcoming underscores that probing the Wnt/β-catenin pathway rather than one of its target genes (Bcl-xL) may have better outcomes in HIV cure strategies. Indeed, we recently demonstrated that inhibiting the β-catenin pathway reactivated HIV not only in a cell model of HIV latency but also from cART-controlled HIV donors and in CD4+ latent cell lines [41]. The translation of our findings in the context of HIV therapy warrants further investigation. The long-term expression of β-catenin to suppress HIV replication (block and lock) maybe risky due to the diverse cellular functions of β-catenin and its association with cell survival and cancer, although, under certain conditions, the activation of the Wnt/β-catenin pathway suppresses malignancy, highlighting the multifaceted functions of β-catenin in disease [42]. To date, Wnt/β-catenin modulators used in clinical trials other than HIV have shown promising results with limited side effects [43,44], underscoring the possibility of exploring this pathway in HIV cure strategies to eliminate the long-lived HIV reservoir.

## Data Availability

The data presented in this study are available on request from the corresponding author.

## Supplementary Materials

The following supporting information can be downloaded at: https://www.mdpi.com/article/10.3390/v14071469/s1, Figure S1: HIV infection induces downregulation of CD4+ T cells and upregulation of CD8+ and CD4^dim^CD8^bright^ T cells; Figure S2: HIV infection induces late apoptosis mediated cell death in CD3+, CD4+ and CD4^dim^CD8^bright^ T cells; Figure S3: Induction of Wnt/β-catenin pathway protects T cell subsets from HIV-mediated early and late apoptosis.
