# Supplementary material for: Wnt/β-Catenin Protects Lymphocytes from HIV-Mediated Apoptosis via Induction of Bcl-xL
Source: Viruses. 2022 Jul 2;14(7):1469. doi: 10.3390/v14071469 (PMC9324643; doi:10.3390/v14071469)

Supp. Figure S1. ns denotes no significance.

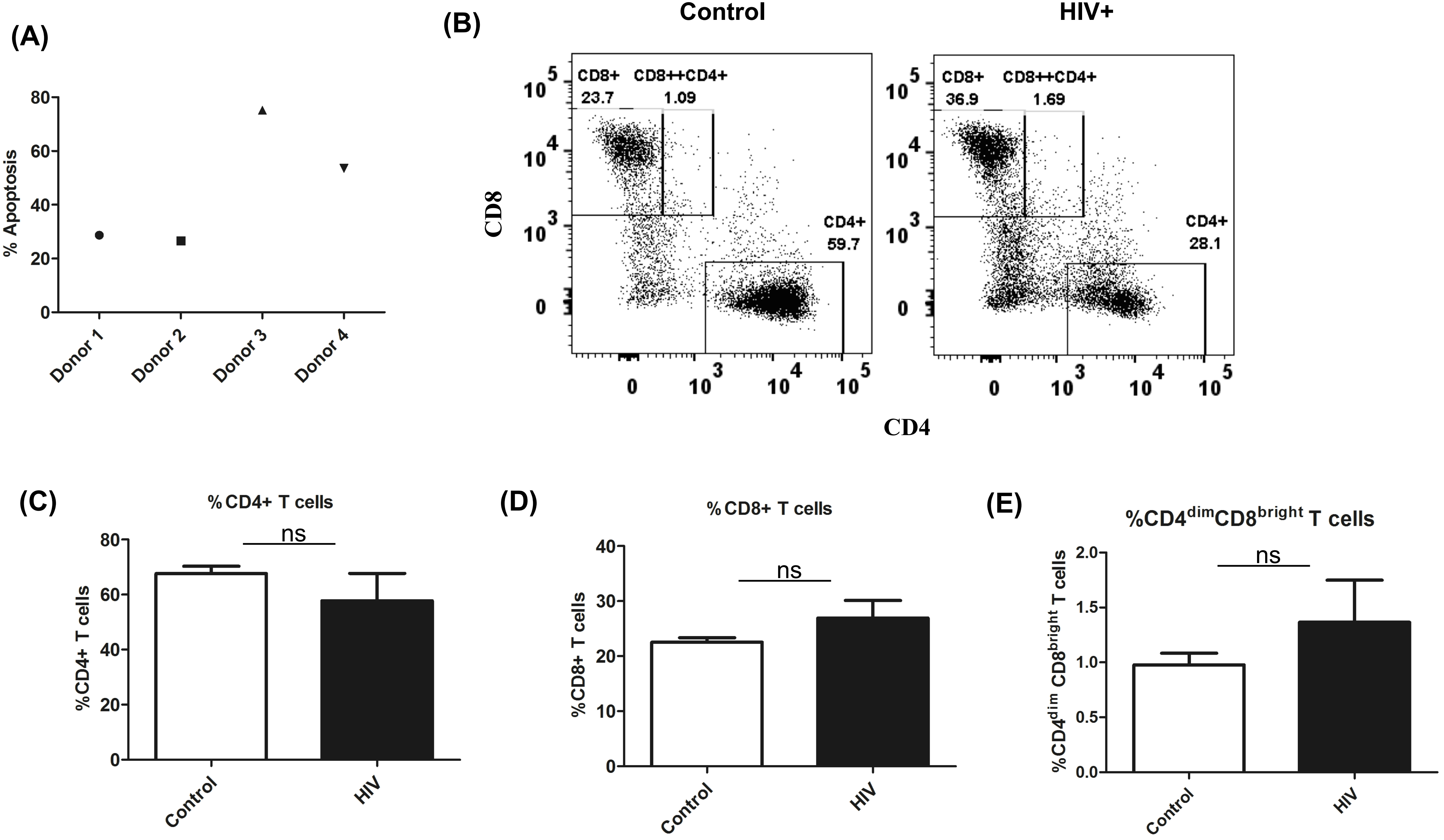

Supp. Figure S2. * means p ≤ 0.05. ns denotes no significance.

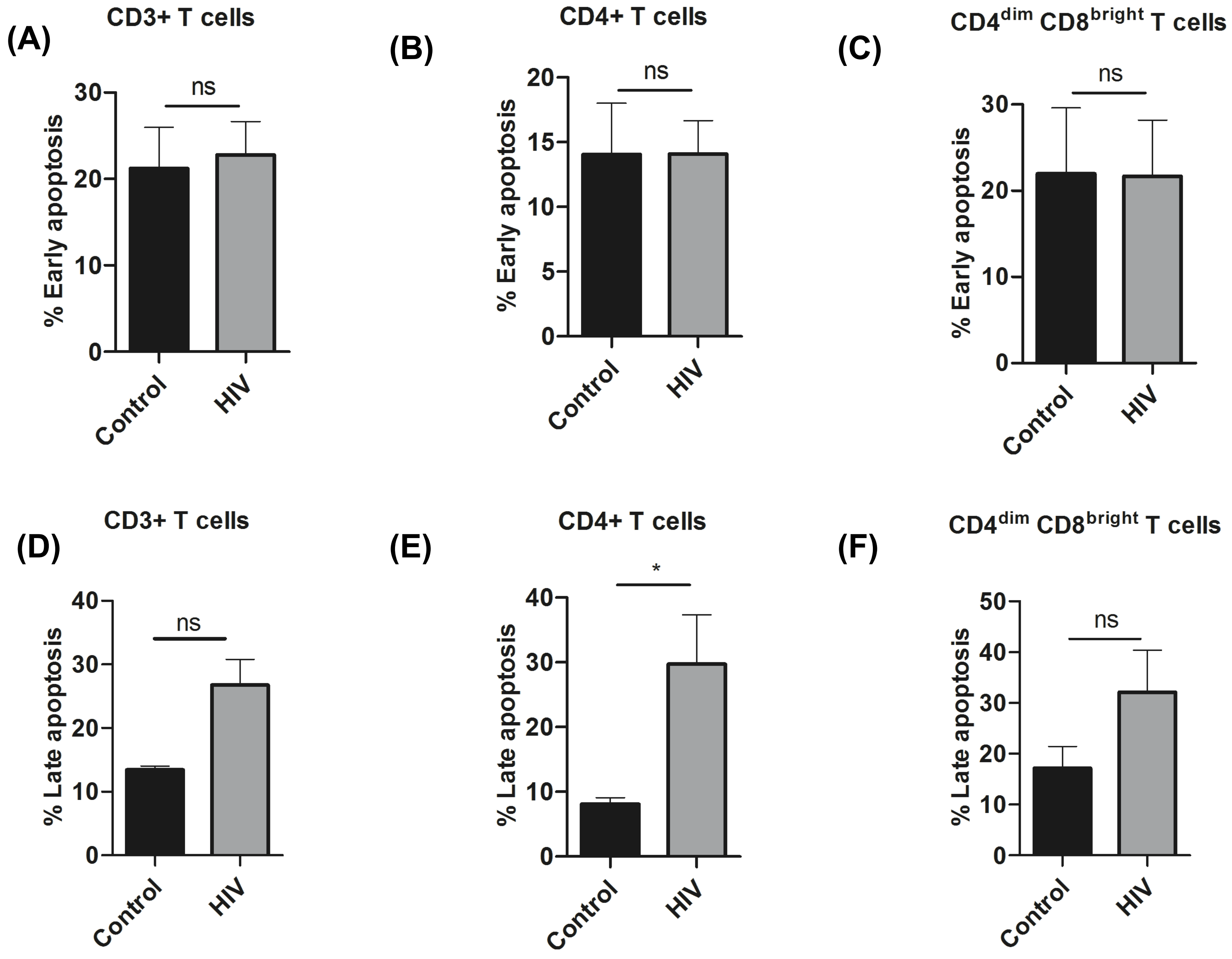

Supp. Figure S3. * means p ≤ 0.05; ** p ≤ 0.01; ns denotes no significance.

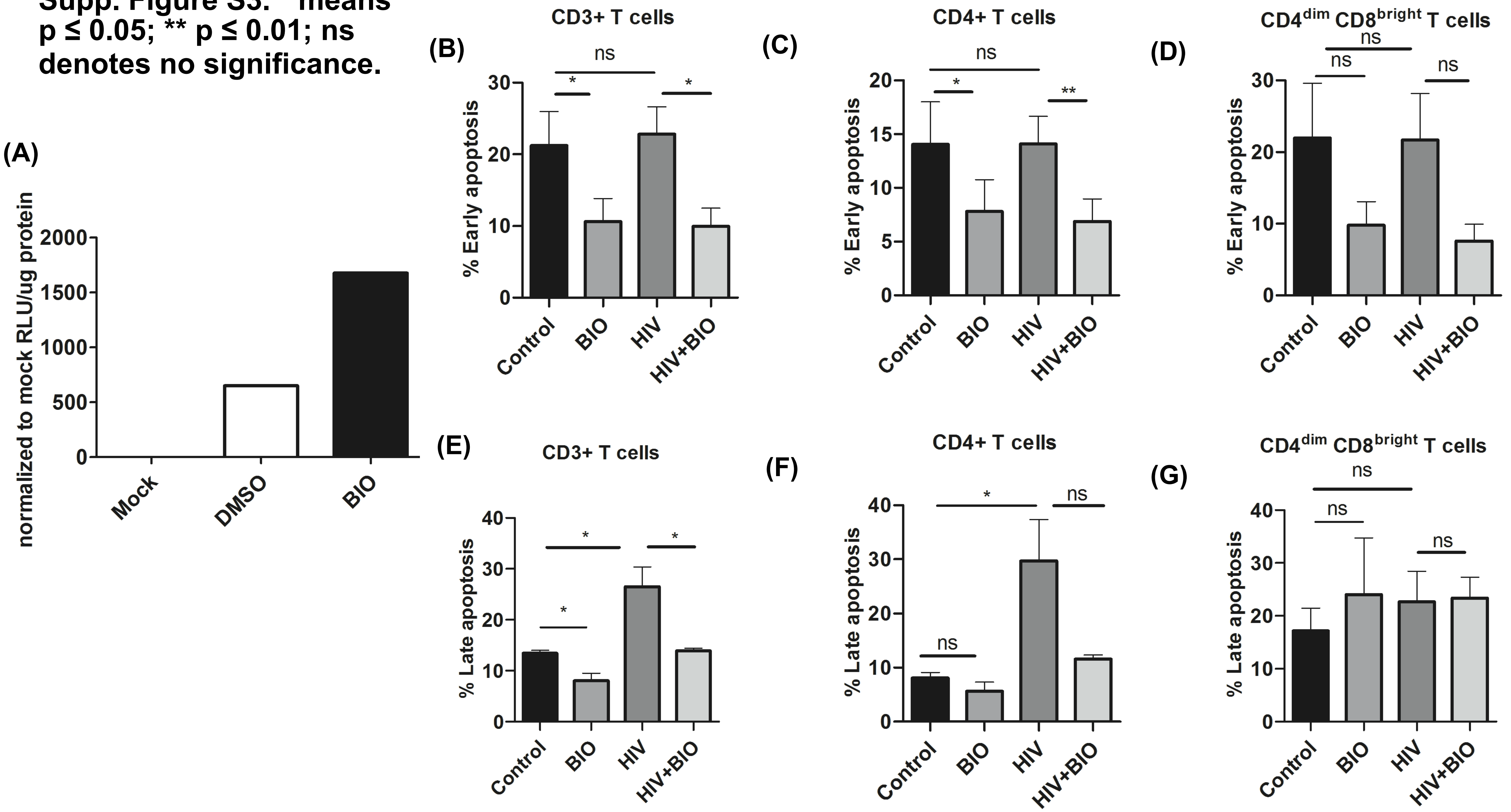

Supplement: Supplementary file 1 [file viruses-14-01469-s001.zip › viruses-1736554-SI.pdf]
